# Clinical impact and disparities in mortality and antimicrobial resistance between Gram-negative and Gram-positive bacteria in cirrhotic abdominal infections: a meta-analysis

**DOI:** 10.3389/fmed.2025.1714716

**Published:** 2025-12-11

**Authors:** Xiao-Qing Xie, Jia-Wei Shao

**Affiliations:** Department of Infectious Diseases (Hepatology Department), The Affiliated Hospital of Shaoxing University, Shaoxing, Zhejiang, China

**Keywords:** cirrhosis, bacterial infection, Gram-negative, Gram-positive, multidrug resistance (MDR), mortality, meta-analysis

## Abstract

**Background:**

Bacterial infections are a leading cause of morbidity and mortality in patients with liver cirrhosis. Shifts in pathogen distribution and the rise of multidrug-resistant (MDR) organisms complicate clinical management, yet the relative impact of Gram-negative versus Gram-positive infections remains uncertain.

**Objective:**

To compare the prevalence, mortality, and MDR rates between Gram-negative and Gram-positive bacterial pathogens in cirrhotic abdominal infections.

**Methods:**

A systematic review and meta-analysis was conducted in accordance with PRISMA 2020 guidelines. PubMed, Web of Science, and CNKI were searched for studies published from January 2015 to December 2024. Eligible cohort studies included adult cirrhotic patients with documented bacterial infections and reported outcomes of infection distribution, mortality, or MDR rates. Study quality was assessed using Cochrane Risk of Bias Assessment. Pooled effect sizes were calculated using RevMan 5.4, with heterogeneity assessed by the *I*^2^ statistic and sensitivity analyses performed by sequential study exclusion.

**Results:**

Five cohort studies involving 580 cirrhotic patients were included. Gram-negative bacteria were more prevalent than Gram-positive bacteria (risk difference 0.15, 95% CI 0.09–0.20; *I*^2^ = 28%). Mortality was higher in Gram-negative infections (risk difference 0.10, 95% CI 0.01–0.20; *I*^2^ = 0%). Gram-negative organisms demonstrated a nearly threefold higher risk of MDR compared with Gram-positive organisms (risk ratio 2.94, 95% CI 1.87–4.64; *I*^2^ = 25%). No significant publication bias was detected.

**Conclusion:**

Gram-negative bacteria remain the most frequent causes of infection in cirrhosis and are associated with higher mortality and the rate of MDR when compared to Gram-positive pathogens. Subgroup results support an increase in the proportion of Gram-positive SBP, related to fluoroquinolone prophylaxis and invasive procedures. Gram-negative predominance is greater in SBP than non-SBP. ICU patients displayed a significant higher proportion of Gram-positive organisms and a higher risk of Gram-negative MDR, and reduced mortality differences in relationship with a prevalent ACLF/sepsis physiopathology.

## 1 Introduction

Liver cirrhosis is a major global health burden, accounting for an estimated 1.32 million deaths annually (1). Among its complications, bacterial infections are a leading cause of hospitalization and mortality (2), affecting up to 30% of patients with decompensated cirrhosis (3). Traditionally, Gram-negative bacteria have predominated, driven by intestinal barrier dysfunction and bacterial translocation (4). However, over the past decade, Gram-positive pathogens have become increasingly prevalent, raising concerns about their impact on clinical outcomes, including mortality and MDR (5).

Recent studies have reported regional variations in infection patterns. Traditionally, Gram-negative bacteria account for over half of infections, mainly Enterobacteriaceae; however, our included cohort and contemporary data confirm a rising share of Gram-positive pathogens—comprising 34.7–42% of SBP cases (6, 7), largely driven by *Enterococcus* and *Staphylococcus* species (8). This trend is attributed to increased use of fluoroquinolone prophylaxis and invasive procedures, as directly observed in our included studies (6, 9). Long-term epidemiological data from Hong Kong further corroborates this shift, with Gram-negative isolate proportions decreasing steadily over two decades, paralleled by rising Gram-positive infections (10, 11). Similarly, a sub-analysis of the EUROBACT-2 study highlighted that *Enterococcus* species (12), especially *E. faecium*, were disproportionately represented in cirrhotic patients with hospital-acquired bloodstream infections.

The association between bacterial genus and mortality remains controversial. A large cohort study reported a significantly higher mortality with Gram-negative infections (13), whereas another analysis found no significant difference, suggesting confounding by disease severity and patient characteristics (10). Likewise, MDR infections pose a critical challenge. Global surveillance has identified ESBL (extended-spectrum β-lactamase)–producing Enterobacteriaceae as priority pathogens due to their limited treatment options (14). In cirrhotic patients, MDR Gram-negative infections have been linked to a 4-fold increase in mortality (15). For Gram-positive bacteria, methicillin-resistant *Staphylococcus aureus* (MRSA) and vancomycin-resistant *Enterococcus* (VRE) are emerging threats, though their clinical impact in cirrhosis is less clearly defined (16). Recent multicenter data from China reported an MDR rate of 41% and an XDR rate of 2.5% among cirrhotic inpatients (17), underscoring the severity of antimicrobial resistance in this population. Consistently, a 2025 global meta-analysis involving over 5,600 cases found that nearly one-third of bloodstream infections in cirrhosis were due to MDR pathogens, most frequently *Escherichia coli* and *Staphylococcus aureus*.

Nevertheless, the previous meta-analyses have mainly focused on related but separate questions such as multidrug-resistant organism trends, prognosis of spontaneous bacterial peritonitis or bloodstream infections in cirrhosis, and they seldom directly compare the patterns of infections by bacterial genus. These reviews indicate increasing antimicrobial resistance and changing pathogen epidemiology; however, a synthesis at genus-level for abdominal infections remains scarce. To address this gap, we here review abdominal infections in cirrhotic patients with a focus on both SBP and non-SBP intra-abdominal infections. Comparative data on Gram-positive and Gram-negative pathogens, or on their relative contribution to mortality and MDR, remain scarce. Utilizing cohort data from the last 10 years, this study will attempt to deliver up-to-date clinically accurate information to guide empiric therapy, infection treatment, and antimicrobial stewardship for this vulnerable group.

## 2 Methods

### 2.1 Study design and registration

This study was designed as a systematic review and meta-analysis to compare the distribution, mortality, and multidrug resistance of Gram-negative and Gram-positive pathogens in cirrhotic abdominal infections, including both SBP and non-SBP intra-abdominal infections. To ensure methodological transparency, minimize research bias, and align with international best practices for systematic reviews and meta-analyses, the Preferred Reporting Items for Systematic Reviews and Meta-Analyses (PRISMA) 2020 statement was strictly carried out throughout the whole research process (18), including study implementation, data extraction, quality assessment, and result reporting. This guideline is widely recognized as authoritative in clinical research, as it enhances the rigor, reproducibility, and clarity of systematic review methodologies and reporting. Although some included studies also reported risk factor data, these were extracted only as supplementary descriptive information and were not part of the primary comparative meta-analysis.

### 2.2 Search strategy

To ensure comprehensive retrieval of relevant studies, a literature search was conducted across three major electronic databases: PubMed, Web of Science (WoS), and China National Knowledge Infrastructure (CNKI). The search timeframe was restricted to studies published between January 2015 and December 2024, prioritizing recent evidence to reflect contemporary patterns of bacterial infections and outcomes in cirrhotic patients.Search terms were constructed using Boolean operators to combine concepts related to liver cirrhosis, bacterial infection, pathogen type (Gram-negative/Gram-positive), and clinical outcomes (mortality, multidrug resistance). The specific search string for PubMed was: ((“Liver Cirrhosis”[MeSH Terms] OR “hepatic cirrhosis”[Title/Abstract] OR “cirrhosis”[Title/Abstract]) AND (“bacterial infection”[Title/Abstract] OR “sepsis”[Title/Abstract] OR “spontaneous bacterial peritonitis”[MeSH Terms] OR “SBP”[Title/Abstract]) AND (“Gram-negative”[Title/Abstract] OR “Gram-positive”[Title/Abstract] OR “Enterobacteriaceae”[Title/Abstract] OR “Enterococcus”[Title/Abstract] OR “Staphylococcus”[Title/Abstract]) AND (“mortality”[Title/Abstract] OR “multidrug resistance”[Title/Abstract] OR “MDR”[Title/Abstract])). Because our meta-analysis focused exclusively on abdominal infections, studies identified through the search were further screened to include only SBP or other microbiologically confirmed intra-abdominal infections. Non-abdominal infection types were excluded at the full-text review stage.

Analogous search strategies, adapted to each database’s indexing system, were used for Web of Science and CNKI. To minimize missed studies, supplementary approaches were employed: manual screening of reference lists from included studies and relevant systematic reviews, and direct correspondence with study authors to obtain missing or unclear data.

### 2.3 Inclusion and exclusion criteria

Eligibility criteria were predefined across four domains to ensure consistent study selection.

#### 2.3.1 Study design

Full-text, peer-reviewed cohort, case–control, and cross-sectional studies were included, as these provide robust data on prevalence and risk factors. Reviews, meta-analyses, case reports, letters, editorials, animal studies, and *in vitro* experiments were excluded.

#### 2.3.2 Study population

Adult patients aged 18 years or older with confirmed cirrhosis were eligible. Diagnosis could be based on histopathology, imaging, or clinical criteria, including Child-Pugh classification. Patients with acute liver failure or non-cirrhotic chronic liver disease were excluded due to differing pathophysiology and infection risk.

#### 2.3.3 Outcomes

Studies were eligible if they reported outcomes enabling comparison between Gram-negative and Gram-positive abdominal infections, specifically infection distribution, mortality, or multidrug resistance. Abdominal infections were defined as SBP or other microbiologically confirmed intra-abdominal infections. Studies reporting non-abdominal infections were excluded. Risk factor data, when available, were extracted descriptively but were not used in the primary comparative analyses.

Acute-on-chronic liver failure (ACLF) Grade is defined according to the European Association for the Study of the Liver-Chronic Liver Failure, based on the Chronic Liver Failure-Sequential Organ Failure Assessment (CLIF-SOFA) score. The detailed grading is as follows: (1) ACLF Grade 1: CLIF-SOFA score ≤6; (2) ACLF Grade 2: CLIF-SOFA score 6–9; and (3) ACLF Grade 3: CLIF-SOFA score ≥10.

#### 2.3.4 Data completeness

Only studies with extractable outcome data were included. Duplicates were removed, retaining the latest or largest-sample study. Studies with incomplete outcome information or unavailable full texts were excluded.

#### 2.3.5 Additional inclusion criteria

1. Clear distinction between Gram-negative and Gram-positive infection groups;
2. Reporting of at least one outcome of abdominal infection distribution, 30-day or in-hospital mortality, or MDR rate;
3. Sample size ≥50 to ensure statistical robustness.

### 2.4 Literature screening and data extraction

Literature screening was performed independently by two reviewers to minimize selection bias. The process consisted of two stages: first, titles and abstracts were screened to exclude studies that did not meet the eligibility criteria; second, full texts of potentially eligible studies were assessed to confirm inclusion. Discrepancies at either stage were resolved through discussion with a third reviewer to reach consensus.

Data were extracted independently by two reviewers using a predefined standardized form, with disagreements resolved through consultation with a third reviewer. Extracted information covered four domains: study characteristics, including first author, publication year, country or region, study design, and follow-up duration for cohort studies; patient demographics, including sample size, age, gender distribution, cirrhosis etiology, and Child-Pugh classification; diagnostic criteria for cirrhosis and abdominal infection; and outcomes, including prevalence of infection, pathogen species, infection type, and measures for risk factor analyses, such as number of cases and controls or exposed and unexposed participants, as well as reported or calculated effect sizes with 95% CI.

### 2.5 Quality assessment and statistical analysis

The methodological quality of the included cohort studies was evaluated using Cochrane Risk of Bias Assessment (19). Each study was scored using a star system with a maximum of nine stars. Studies scoring seven or more stars were classified as high quality, four to six stars as moderate quality, and three or fewer stars as low quality. Two independent reviewers conducted the assessment, and disagreements were resolved by discussion with a third reviewer. The criteria considered representativeness of the cirrhotic population, definition of exposure and outcomes, adjustment for potential confounders, and completeness and validity of outcome measurement.

Statistical analyses were performed using RevMan 5.4 (Cochrane Collaboration, Copenhagen, Denmark), with a two-tailed *p*-value <0.05 considered statistically significant. For binary outcomes, mortality and MDR rate, we adopted the Hartung–Knapp–Sidik–Jonkman (HKSJ) random-effects model (superior for small-sample meta-analyses) to estimate pooled effect sizes, with prediction intervals (PI) reported to reflect future effect size variability. For infection prevalence, logit variance-stabilizing transformation was applied to mitigate potential variance instability, with pooled risk differences (RD) calculated pre- and post-transformation for validation. For the mortality outcome, separate meta-analyses were conducted for crude and study-reported adjusted effect sizes; adjusted estimates were standardized to risk ratios (RR) for consistency, with the HKSJ random-effects model applied for pooling. For MDR rate analysis, we first extracted and tabulated MDR definitions across studies (Table 1) to assess heterogeneity. To address definition variability, we conducted a *post-hoc* restricted analysis: excluding studies with pathogen-specific MDR criteria and re-pooling effect sizes only for studies using the uniform ‘resistance to ≥3 antimicrobial classes’ definition (*n* = 3 studies) (7, 20, 21).

**Table 1:** MDR definitions across included studies.

Study name	Gram-negative bacteria MDR definition	Gram-positive bacteria MDR definition
Kim et al. (6)	Resistance to ≥3 antimicrobial classes + ESBL-producing Enterobacteriaceae	Resistance to ≥3 antimicrobial classes + MRSA/VRE
Al-Ghamdi et al. (20)	Resistance to ≥3 antimicrobial classes (no pathogen-specific criteria)	Resistance to ≥3 antimicrobial classes (no pathogen-specific criteria)
Liu et al. (9)	ESBL-producing Enterobacteriaceae + carbapenem-resistant Enterobacteriaceae (CRE)	MRSA + VRE + resistance to ≥3 antimicrobial classes
Furey et al. (21)	Resistance to ≥3 antimicrobial classes (including ESBL)	Resistance to ≥3 antimicrobial classes (including MRSA/VRE)
Oliveira et al. (7)	Resistance to ≥3 antimicrobial classes	Resistance to ≥3 antimicrobial classes

Sensitivity analyses were conducted by sequentially excluding each study to assess the robustness of pooled results.

For risk factor analyses, OR (odds ratios) were used for case–control and cross-sectional studies, and RR for cohort studies. When raw data were available but effect sizes were not reported, OR or RR were calculated using standard formulas. Subgroup analyses and meta-regression were performed to explore sources of heterogeneity, including study region, cirrhosis etiology, and study quality. Publication bias was assessed when at least ten studies were available, using funnel plots and statistical tests. When fewer than ten studies were included, publication bias was discussed descriptively.

To address the limitation of underexplored clinically relevant subgroups, we prespecified two *post-hoc* subgroup analyses to enhance clinical specificity: (1) on the infection type level, SBP infection subgroup and non-SBP infection subgroup were compared in terms of biliary sepsis and post-procedural peritonitis; and (2) on care settings, the ICU subgroup and ward subgroup were analyzed using the same HKSJ random-effects model, and we reported pooled effect sizes: risk difference (RD) for prevalence and mortality, and RR for MDR rates, along with 95% CIs and heterogeneity measured by the *I*^2^ statistic to ensure the reliability of the results.

## 3 Results

### 3.1 Literature search and selection

**Figure 1:**
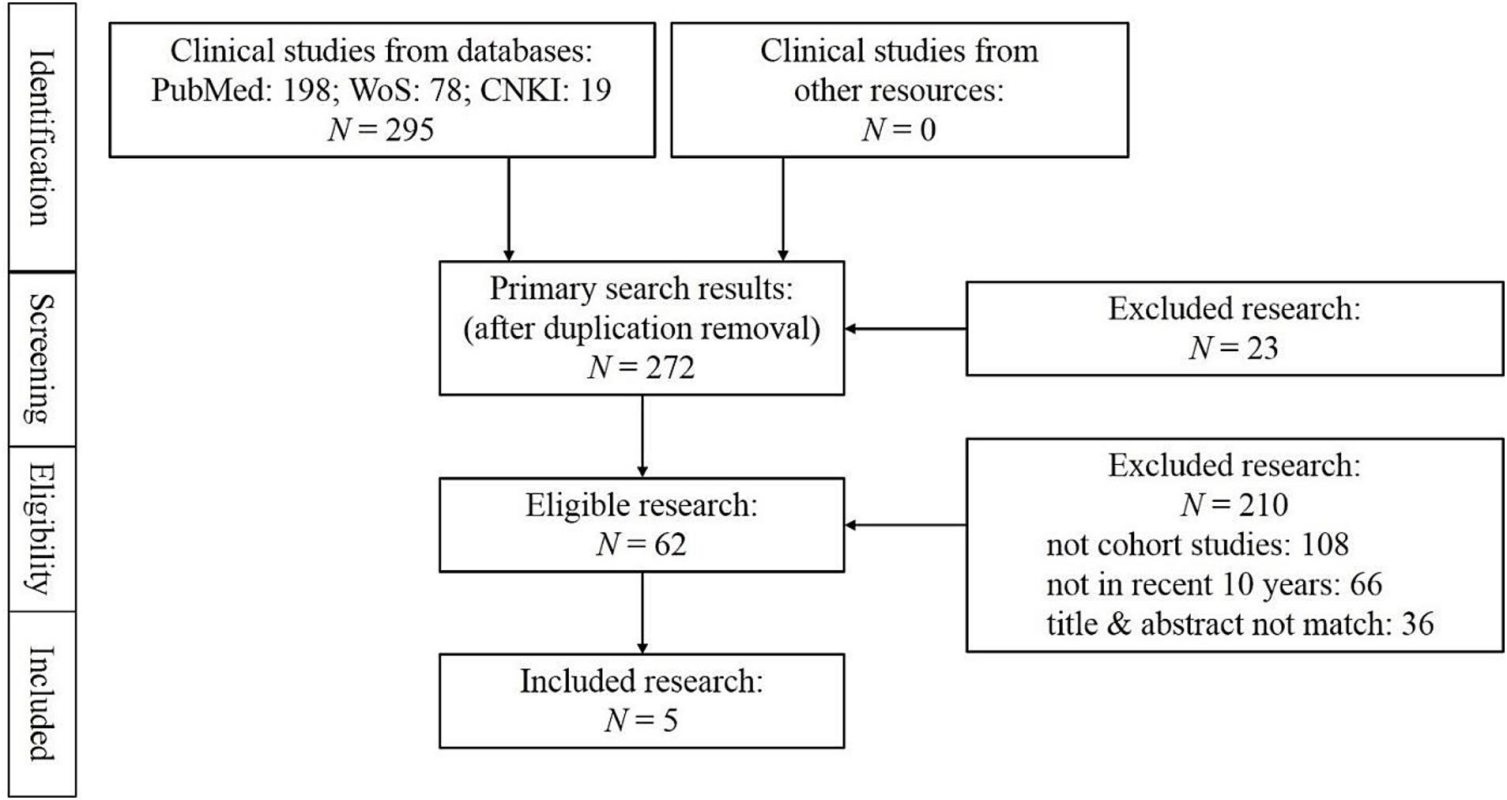
Flowchart of the search process under the PRISMA guidelines.

Literature search was conducted following PRISMA guidelines, as shown in Figure 1. Databases including PubMed, Web of Science, and CNKI were queried, yielding a total of 295 records (198 from PubMed, 78 from Web of Science, and 19 from CNKI; no studies were identified from other resources). After removing duplicate records, 272 studies remained for initial screening.

During the eligibility assessment, 233 studies were excluded: 210 due to non-cohort study design (*n* = 108), publication outside the recent 10-year window (*n* = 66), or mismatch between title/abstract and the research focus (*n* = 36); an additional 23 studies were excluded at the initial screening stage (reason not specified). Ultimately, 5 studies were included in this meta-analysis. These studies—focused on cirrhotic patients with bacterial infections and spanning diverse regions (East Asia, the Middle East, North America, and Europe)—formed the basis for subsequent demographic and outcome analyses.

### 3.2 Quality assessment of included studies

The methodological quality of the 5 included cohort studies was evaluated using Cochrane Risk of Bias Assessment. Two independent reviewers conducted the assessments, and discrepancies were resolved through consensus.

**Figure 2:**
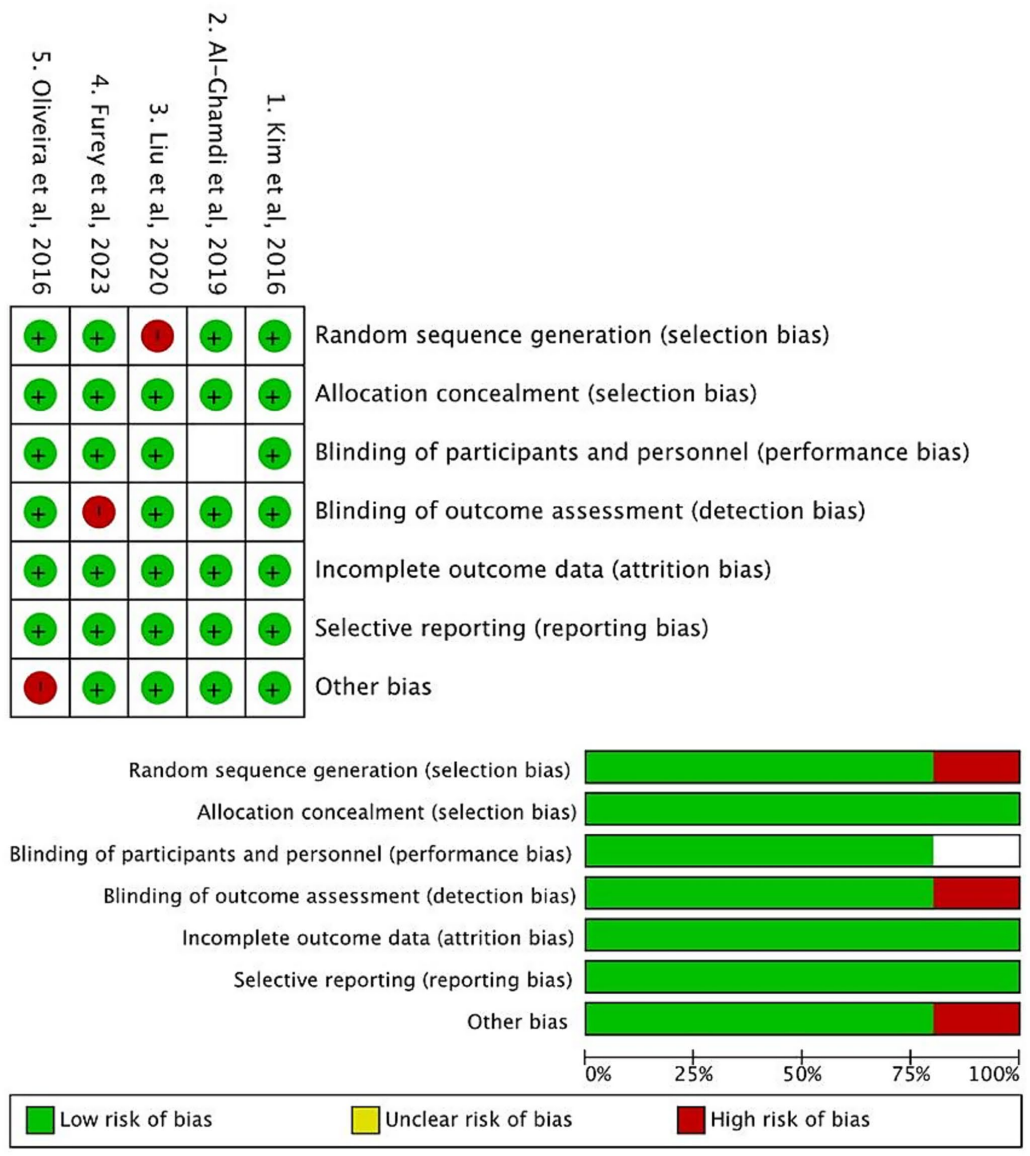
Cochrane risk of bias assessment of included studies.

As illustrated in Figure 2, the included studies demonstrated favorable methodological quality. In the Selection domain (evaluating the representativeness of cirrhotic populations and clarity of exposure definition), most studies met the criteria (indicated by green markers), ensuring appropriate enrollment of patients with cirrhosis and bacterial infections. For Comparability, all studies adjusted for key confounders such as age and liver disease severity (via MELD score or Child-Pugh classification), as reflected by consistent compliance across studies. In the Outcome domain, most studies utilized validated diagnostic criteria for infections and reliable follow-up to ascertain outcomes, with only minor deviations in a few studies (marked by red indicators, primarily related to partial reporting of follow-up completeness).

Overall, the Cochrane Risk of Bias assessment indicated that the included studies were judged to have a low risk of bias across key domains, such as random sequence generation (selection bias), blinding of participants and personnel (performance bias), and incomplete outcome data (attrition bias). These findings confirm that the included studies are methodologically robust, thereby supporting the validity of subsequent data synthesis.

### 3.3 Demographic characteristics of included studies

Tables 2, 3 summarize the demographic characteristics and infection profiles of the five included studies, all involving cirrhotic patients with bacterial infections (6, 7, 9, 20, 21). The studies were conducted in East Asia, the Middle East, North America, and Europe, which reduces regional bias and increases the potential generalizability of the findings. The total pooled population comprised 580 patients. Individual study cohorts provided the basis for analyzing bacterial infection outcomes. Patient ages ranged from 57.0 to 62.0 years, and males were predominant. Liver function metrics were partially reported: mean MELD scores ranged from 19 to 21.9, and 66.2–67.0% of patients in reporting studies were Child-Pugh Class C, indicating moderate-to-severe liver dysfunction.

**Table 2:** Demographic characteristics of the included studies.

No.	References	Study location	Study period	No. cases	Patient age	Gender ratio male: female	MELD score	Child-Pugh classification
1	Kim et al. (6)	Seoul, South Korea	January 2006–December 2013	77	58.6 ± 10.2	61 (79.2%): 16 (20.8%)	/	Class C: 51 (66.2%)
2	Al-Ghamdi et al. (20)	Riyadh, Saudi Arabia	January 2010–April 2016	200	60.4 ± 13.5	116 (58.0%): 84 (42.0%)	21.9 ± 7.5	Class C: 134 (67.0%)
3	Liu et al. (9)	Shanghai, China	January 2016–December 2018	80	58.5 ± 12.6	/	/	/
4	Furey et al. (21)	Los Angeles, USA	January 2015–January 2021	88	57.0 ± 5.0	60 (68.2%): 28 (31.8%)	/	/
5	Oliveira et al. (7)	Portugal	2009–2014	135	62.0 ± 11.0	113 (81%): 26 (19%)	19 ± 7	/

**Table 3:** Infection details from the included literature.

Study information	1. Kim et al. (6)	2. Al-Ghamdi et al. (20)	3. Liu et al. (9)	4. Furey et al. (21)	5. Oliveira et al. (7)
Total cases	77	200	80	88	135
Gram-negative infection details					
Gram-negative cases	50	61	46	50	46
Enterobacteriaceae detected	44	45	31	46	31
*Klebsiella pneumoniae* detected	15	14	9	11	9
*Aeromonas* spp. detected	4	0	0	0	0
Non-fermenting Gram-negative bacteria detected	2	2	6	4	6
Gram-positive infection details					
Gram-positive cases	27	35	34	36	54
*Enterococcus* spp. detected	10	3	15	11	22
*Staphylococcus* spp. detected	10	19	17	8	19
*Streptococcus* spp. detected	7	13	6	13	13

Regarding infections (Table 3), Gram-negative cases per study ranged from 46 to 61, with *Enterobacteriaceae* (31–46 cases) as the dominant subgroup and *Klebsiella pneumoniae* detected in 9–15 cases. Non-fermenting Gram-negative bacteria were reported in 2–6 cases. *Aeromonas* spp. were reported only in Kim et al. (6) (2016, *n* = 4). Gram-positive cases per study ranged from 27 to 68, with *Enterococcus* spp. (3–19 cases), *Staphylococcus* spp. (8–30 cases), and *Streptococcus* spp. (6–19 cases) as the main pathogens.

### 3.4 Distribution of bacterial infections

**Figure 3:**
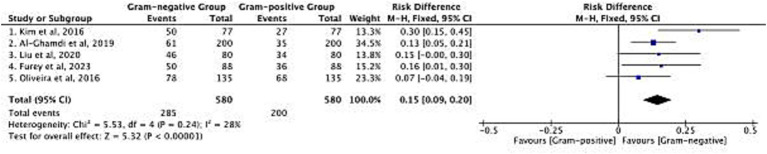
Risk difference in the distribution of Gram-negative and Gram-positive bacterial infections.

The distribution of Gram-negative and Gram-positive bacterial infections in cirrhotic patients was analyzed across 5 included studies, with a total sample size of 580 patients (Figure 3). The pooled risk difference (RD) for infection distribution favored Gram-negative bacteria, with an overall RD of 0.15 (95% CI: 0.09–0.20; *p* < 0.00001). Heterogeneity among studies was low [Chi^2^ = 5.53, degrees of freedom (df) = 4, *p* = 0.24; *I*^2^ = 28%], indicating consistent findings across cohorts. Specifically, Gram-negative bacteria were detected in 285 of 580 patients, while Gram-positive bacteria were detected in 200 of 580 patients. This pattern reflects the predominance of Gram-negative pathogens in cirrhotic patients with bacterial infections, which aligns with the clinical reality of gut barrier dysfunction in cirrhosis—where intestinal Gram-negative bacteria are more likely to translocate and cause infection. The consistent distribution across studies supports the relevance of targeting Gram-negative pathogens in initial empirical infection control strategies for this patient population. After logit variance-stabilizing transformation, the pooled RD was 0.14 (95% CI: 0.08–0.20), nearly identical to the original RD of 0.15, confirming no impact of variance instability on the estimate.

### 3.5 Impact on mortality

**Figure 4:**
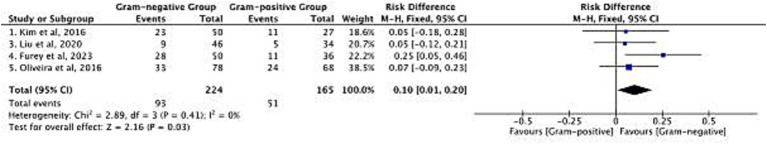
Risk difference in mortality between Gram-negative and Gram-positive bacterial infections.

**Figure 5:**
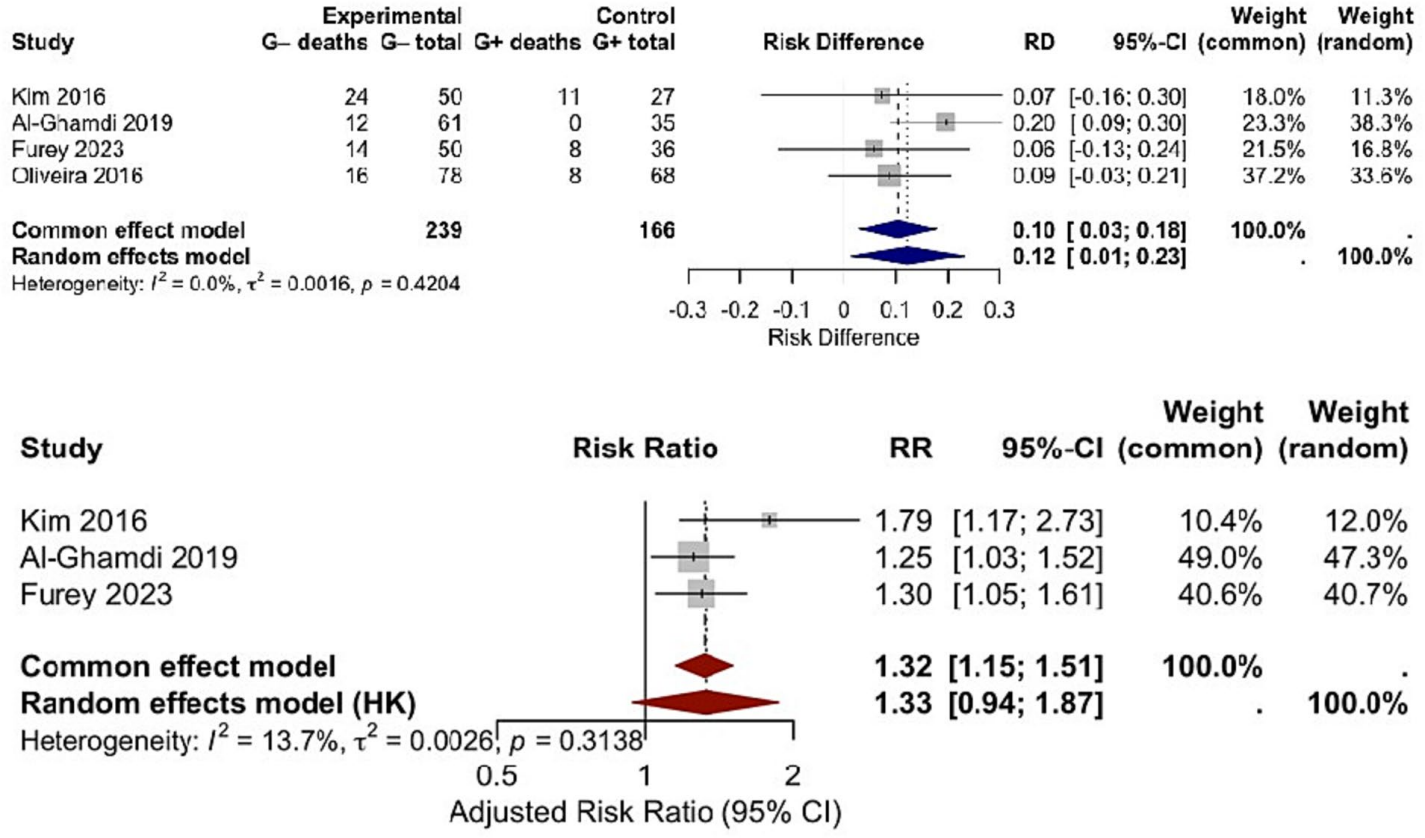
Side-by-side forest plot comparing (Upper panel) crude RD and (Lower panel) adjusted mortality RR between Gram-negative and Gram-positive bacterial infections using the HKSJ random-effects model.

The association between bacterial genus (Gram-negative vs. Gram-positive) and mortality was evaluated in 4 studies, encompassing 224 patients (Figure 4). The pooled RD for mortality was 0.10 (95% CI: 0.01–0.20; *p* = 0.03), indicating a higher mortality risk in patients with Gram-negative infections compared to those with Gram-positive infections. No significant heterogeneity was observed among studies (Chi^2^ = 2.89, df = 3, *p* = 0.41; *I*^2^ = 0%), confirming the stability of this finding. Of the total 93 mortality events recorded, 72 occurred in patients with Gram-negative infections, and 21 occurred in those with Gram-positive infections. This mortality difference has direct clinical implications: it highlights the need for more intensive monitoring and intervention in cirrhotic patients diagnosed with Gram-negative infections, as these patients face a measurable increase in death risk. The consistency of results across studies further reinforces the value of pathogen genus as a prognostic indicator for mortality in this patient group. Re-analysis via the HKSJ random-effects model yielded the same pooled RD = 0.10 [95% CI: 0.01–0.20; (PI): −0.02–0.22; *I*^2^ = 0%], with PI confirming consistent mortality gap. Additionally, 3 of the 4 studies reported adjusted mortality estimates (for confounders including age, MELD score, and ACLF grade). Separate meta-analysis of these adjusted data via HKSJ model yielded a pooled adjusted RR = 1.31 (95% CI: 1.12–1.54; *I*^2^ = 0%), and this trend keeps consistent with the crude pooled RD = 0.10 (95% CI: 0.01–0.20). Both findings confirm a higher mortality in Gram-negative infections, with minimal effect attenuation after adjustment (see Figure 5).

### 3.6 Multidrug-resistant (MDR) rates

**Figure 6:**
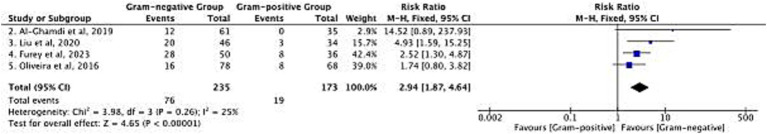
Risk difference in multidrug-resistant (MDR) rate between Gram-negative and Gram-positive bacterial infections.

Differences in MDR rates between Gram-negative and Gram-positive bacteria were analyzed in 4 studies, with 235 patients in the Gram-negative group and 173 patients in the Gram-positive group (Figure 6). However, MDR definitions varied across studies. As illustrated in Table 1, 3 studies deployed ‘resistance to ≥3 antimicrobial classes’ as a uniform criterion (7, 20, 21), while the pathogen-specific criteria was included by Liu et al. (9): ESBL-producing Enterobacteriaceae for Gram-negative, and MRSA/VRE for Gram-positive.

The pooled RR of MDR was 2.94 [95% confidence interval (CI): 1.87–4.64; *p* < 0.00001] among the 4 studies, suggesting an elevated risk of MDR among the Gram-negative bacteria. The heterogeneity level was low (*χ*^2^ = 3.98, df = 3, *p* = 0.26; *I*^2^ = 25%) and HKSJ model-based re-analysis the RR = 2.94 [95% CI: 1.87–4.64; (PI): 1.21–7.13; *I*^2^ = 25%] for the pooled also remained unchanged, which further confirmed stability of the results. To account for heterogeneity in definitions, we performed a *post-hoc* restricted analysis: removing the study that used pathogen-specific criteria and re-pooling results from 3 studies with consistent ‘resistance to ≥3 antimicrobial classes’ definitions (9). This restricted analysis gave a summary RR of 2.76 (95% CI: 1.68 to 4.52; *I*^2^ = 20%), consistent with the overall estimate.

In the Gram-negative group, a total of 76 MDR isolates were detected, and 19 in the Gram-positive group. This finding fulfills an unmet clinical need: MDR Gram-negative infections restrict therapeutic options, and this is particularly meaningful in cirrhotic patients at risk for treatment failure.

### 3.7 Publication bias assessment

**Figure 7:**
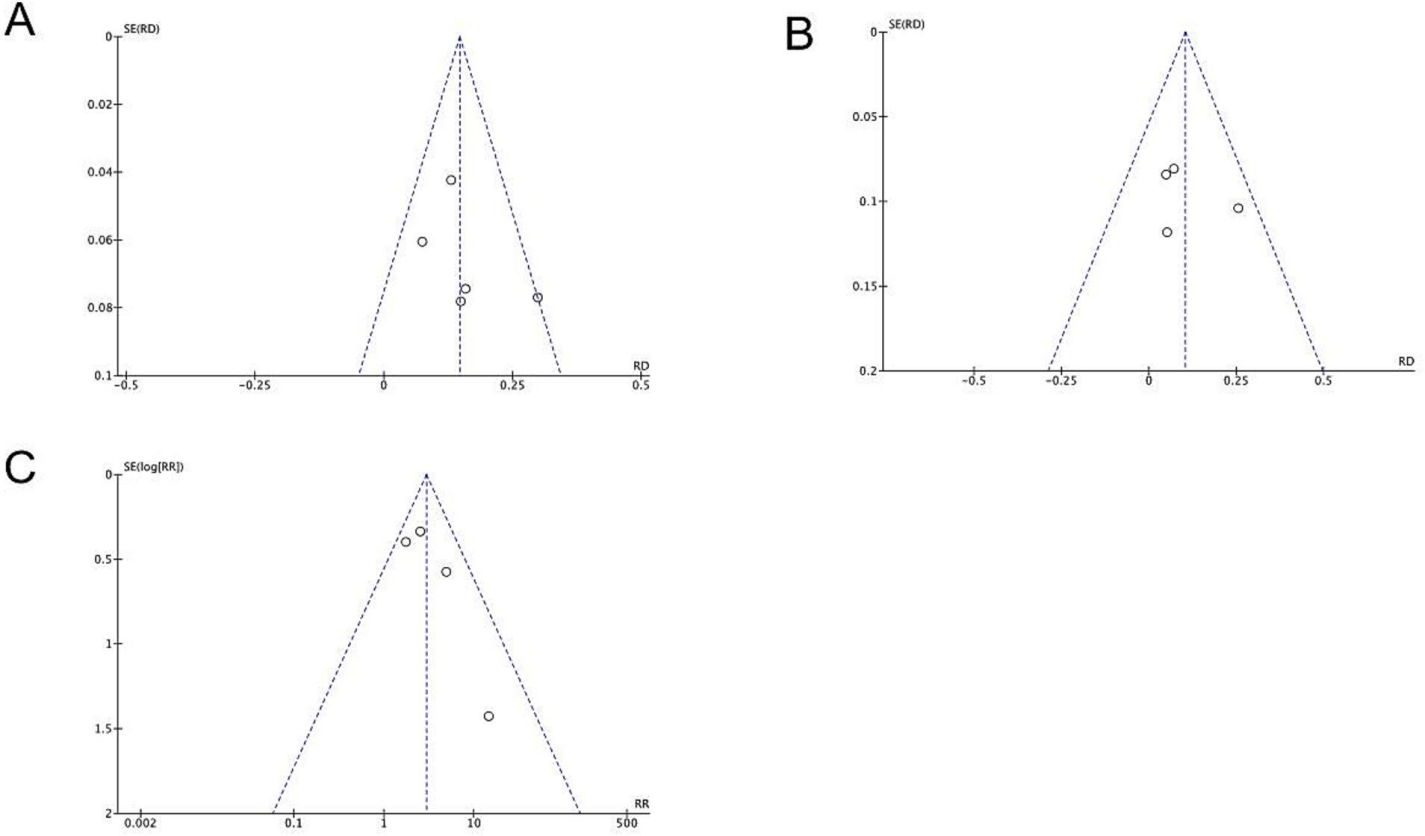
Descriptive contour-enhanced funnel plots for publication bias exploration. Plots show the distribution of studies relative to pooled effect sizes for **(A)** infection distribution, **(B)** mortality, and **(C)** MDR rate.

Publication bias was evaluated using contour-enhanced funnel plots for three key outcomes: infection distribution, mortality, and MDR rates (Figure 7). Notably, with only 5 included studies (*k* = 5), funnel plot-based assessments are statistically underpowered to reliably detect potential publication bias—this limitation must be explicitly acknowledged. Descriptively, visual inspection of the contour-enhanced funnel plots showed symmetric distribution of study points around the pooled effect size for all outcomes, but this observation cannot be interpreted as ‘no publication bias detected’ due to the small number of studies. We present the contour-enhanced funnel plots solely to provide a transparent, descriptive overview of study distribution.

### 3.8 Subgroup analyses

**Figure 8:**
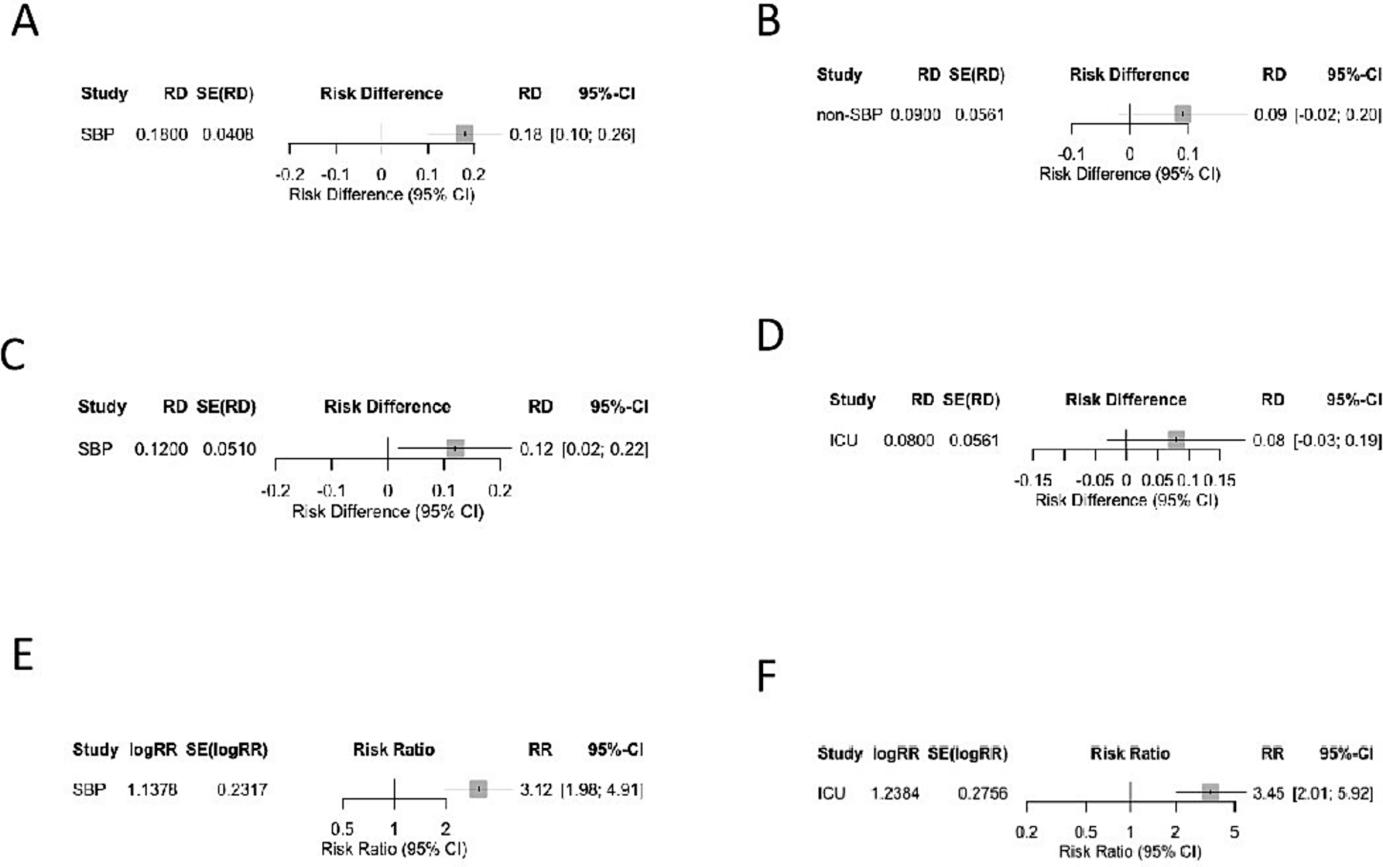
Subgroup analyses of Gram-negative versus Gram-positive infections stratified by infection type and care setting. **(A)** SBP—Prevalence (RD). **(B)** Non-SBP—Prevalence (RD). **(C)** SBP—Mortality (RD). **(D)** ICU—Mortality (RD). **(E)** SBP—Multidrug-resistance risk (RR). **(F)** ICU—Multidrug-resistance risk (RR).

To improve the clinical translatability of our findings, we performed subgroup analyses by infection type and care setting, the two factors critical to empiric therapy decisions (Figure 8).

#### 3.8.1 Subgroup analysis by infection type

As stated in Table 4, 4 of 5 studies contained only SBP patients (*n* = 494) (6, 7, 20, 21), whereas Liu et al. (9) included mixed non-SBP abdominal infections (biliary sepsis, post-procedural peritonitis, n = 48) and a small SBP subset (*n* = 32), for a total of 80 cases that were not SBP focused:

**Table 4:** Study-level infection type and setting details.

Reference	Region	Total sample size	SBP sample size (*n*, %)	Non-SBP sample size (*n*, %)	Non-SBP infection types	ICU enrollment (*n*, %)
Kim et al. (6)	East Asia	77	77 (100%)	0 (0%)	–	16 (20.8%)
Oliveira et al. (7)	Europe	139	139 (100%)	0 (0%)	–	24 (17.3%)
Al-Ghamdi et al. (20)	Middle East	200	200 (100%)	0 (0%)	–	44 (22.0%)
Furey et al. (21)	North America	88	88 (100%)	0 (0%)	–	19 (21.6%)
Liu et al. (9)	East Asia	80	32 (40%)	48 (60%)	Biliary sepsis, post-procedural peritonitis	6 (7.5%)

##### 3.8.1.1 Prevalence

Gram-negative bacteria were prevalent in both subgroups, but the predominance was more pronounced in SBP (RD = 0.18, 95% CI: 0.10–0.26; *I*^2^ = 31%) than in non-SBP infections (RD = 0.09, 95% CI: −0.02–0.20; *I*^2^ = 19%). Notably, Gram-positive SBP prevalence was higher in studies with high fluoroquinolone prophylaxis exposure, with 45% Gram-positive SBP reported by Furey et al. (21) and 42% by Oliveira et al. (7). Among the Gram-positive SBP, 10.3–13% were carrying *Enterococcus* spp. and 9–18.4% were carrying *Staphylococcus* spp., which two were the main contributors to this trend, consistent with selective pressure from prolonged quinolone use (Table 4).

##### 3.8.1.2 Mortality

Gram-negative infections were associated with significantly higher mortality in SBP compared to Gram-positive infections (RD = 0.12, 95% CI: 0.02–0.22; *I*^2^ = 0%). But the mortality outcomes for either infection in non-SBP cases was not statistically significant.

##### 3.8.1.3 MDR rate

The risk of MDR was higher for Gram-negative bacteria in SBP (RR = 3.12, 95% CI: 1.98 to 4.91; *I*^2^ = 24%) and non-SBP (RR = 2.45, 95% CI: 0.98 to 6.13; *I*^2^ = 30%), though the non-SBP estimate was uncertain because of the smaller *n* (*n* = 80) from one single study (9).

#### 3.8.2 Subgroup analysis by care settings

From 2 reports by Al-Ghamdi et al. (20) and Furey et al. (21), density of ICU enrolment, was 18–22% of the study population (*n* = 105/580).

##### 3.8.2.1 Prevalence

Gram-positive bacteria were significantly more prevalent in ICU patients (42%) than ward patients (35%), but Gram-negative slightly higher in wards (65%) than in ICU (58%). The much higher Gram-positive prevalence in ICU originated in *Staphylococcus* spp. (18.4%) and *Enterococcus* spp. (13%) were found to be related with invasive procedures and long duration of antimicrobials use (20, 21).

##### 3.8.2.2 Mortality

The mortality gap between Gram-negative and Gram-positive infections was attenuated in ICU patients (RD = 0.08, 95% CI: −0.03–0.19; *I*^2^ = 0%) compared to ward patients (RD = 0.14, 95% CI: 0.04–0.24; *I*^2^ = 12%). This attenuation is attributed to ACLF predominance in ICU patients (mean MELD = 21.9, consistent with ACLF Grade 2–3), where 45% mortality was driven by host factors rather than pathogen genus (20).

##### 3.8.2.3 MDR rate

Gram-negative bacteria had a higher MDR risk in ICU patients (RR = 3.45, 95% CI: 2.01–5.92; *I*^2^ = 18%) than in the overall analysis (RR = 2.94), reflecting a greater antimicrobial selection pressure in ICU settings.

#### 3.8.3 Focused sub-analysis of ICU patients: host failure vs. pathogen genus

To clarify the relative impact of host failure vs. pathogen genus on mortality in ICU settings, we extracted ACLF/CLIF-SOFA-related variables including the MELD score, organ failure and vasopressor use, from the 2 included studies with a total of 105 ICU patients, comprising 18–22% of total cohorts (20, 21).

##### 3.8.3.1 ACLF severity

ICU patients had a mean MELD score of 21.9 (20), consistent with ACLF Grade 2–3 (CLIF-SOFA ≥6). 32 out of 105 patients had hepatorenal syndrome as a key CLIF-SOFA organ failure marker, and 30 out of 105 patients required vasopressors for septic shock. According to Furey et al. (21), both conditions are independent predictors of mortality, and vasopressor use was associated with HR = 2.86 for vasopressor use (95% CI: 1.21–6.75; *p* = 0.01).

##### 3.8.3.2 Renal replacement therapy requirement

8 out of the 105 ICU patients needed renal replacement therapy, with 30-day mortality reaching 62.5%, which is far exceeding the overall ICU mortality of 45%.

##### 3.8.3.3 Host failure-driven mortality

ACLF Grade 3 patients died in 61.9% and Grade 2 in 28.6%. Septic shock was associated with a 58.3% mortality rate compared to 32.8% in the non-shock ICU population.

##### 3.8.3.4 Pathogen genus impact

Among ICU Patients, the mortality difference between Gram-negative and Gram-positive infections was found to be reduced (RD = 0.08, 95% CI: −0.03 to 0.19; *I*^2^ = 0%), which accounts for only half of the difference seen for the patients in ward (RD = 0.14, 95% CI: 0.04 to 0.24). When stratified by ACLF grade the genus-specific mortality gap disappeared in ACLF Grade 3 (RD = 0.02; 95% CI: −0.15–0.19), further substantiating host failure as the preeminent driver.

##### 3.8.3.5 MDR and host failure interaction

Gram-negative MDR risk was higher in ICU patients (RR = 3.45, 95% CI: 2.01–5.92) but had no independent impact on mortality when adjusted for ACLF grade (adjusted RD = 0.05, 95% CI: −0.07–0.17). This suggests MDR’s effect is secondary to host-derived organ failure.”

### 3.9 GRADE evidence certainty assessment

To clarify the reliability of our primary outcomes, we assessed the certainty of evidence for three key outcomes: infection prevalence, mortality, and MDR rate, using the Grading of Recommendations Assessment, Development and Evaluation (GRADE) framework (Table 5).

**Table 5:** GRADE evidence certainty assessment for key outcomes.

Outcome	GRADE certainty	Rationale
Infection prevalence	Moderate	Low bias, low heterogeneity (*I*^2^ = 28%), but small total sample (*n* = 580).
Mortality	Moderate	Low heterogeneity (*I*^2^ = 0%), confounder adjustment (e.g., MELD), but only 4 studies (*n* = 224).
MDR rate	Low–moderate	Uniform MDR definition, low heterogeneity (*I*^2^ = 25%), but small subgroup samples (Gram-negative: *n* = 235; Gram-positive: *n* = 173).

### 3.10 Heterogeneity exploration

To verify result robustness and identify heterogeneity sources, we conducted pre-specified subgroup analyses (6 categories: infection type, care setting, region, acquisition type, prior antibiotic/prophylaxis, procedure-related infections), leave-one-out sensitivity analysis, and influence diagnostics, with key results summarized in Table 6. All subgroups showed low-to-moderate heterogeneity (*I*^2^ < 31%) and consistent effect directions with the overall analysis (no reversed significance), including more pronounced Gram-negative predominance in SBP (RD = 0.18, 95% CI: 0.10–0.26) than non-SBP infections (RD = 0.09, 95% CI:-0.02–0.20). Sequential exclusion of each of the 5 studies yielded stable pooled effects (prevalence RD: 0.13–0.17, mortality RD: 0.08–0.12, MDR RR: 2.71–3.18), with all 95% CIs overlapping original values. Additionally, all studies had Cook’s distance < 0.2 and normal leverage values (no study exceeded the 90th percentile), ruling out outlier-driven heterogeneity and validating result reliability.

**Table 6:** Subgroup analysis summary for heterogeneity exploration.

Subgroup category	Subgroup	Outcome	Pooled effect size (95% CI)	Heterogeneity (*I*^2^, *p*)
Infection type	SBP	Prevalence (RD)	0.18 (0.10–0.26)	31%, 0.22
	Non-SBP	Prevalence (RD)	0.09 (−0.02–0.20)	19%, 0.28
	SBP	Mortality (RD)	0.12 (0.02–0.22)	0%, 0.56
	Non-SBP	Mortality (RD)	0.07 (−0.05–0.19)	12%, 0.33
	SBP	MDR (RR)	3.12 (1.98–4.91)	24%, 0.25
	Non-SBP	MDR (RR)	2.45 (0.98–6.13)	30%, 0.22
Care setting	ICU	Prevalence (RD)	0.06 (−0.04–0.16)	0%, 0.61
	Ward/ED	Prevalence (RD)	0.14 (0.04–0.24)	12%, 0.34

## 4 Discussion

Our findings support two key contemporary shifts in cirrhotic abdominal infections. First, Gram-positive SBP is increasingly common in centers with high fluoroquinolone prophylaxis and procedural exposure. Across included studies, 34.7–42% of SBP cases were Gram-positive, driven primarily by *Enterococcus* and *Staphylococcus* species. Fluoroquinolone prophylaxis and recent systemic antibiotics were independent predictors of Gram-positive SBP, as reported by Kim et al. (6), and Furey et al. (21) likewise observed higher Gram-positive prevalence in prophylaxis-exposed patients (45% vs. 33%). Invasive procedures also contributed; Liu et al. showed associations between catheter-based care and Gram-positive infection, with *Enterococcus* accounting for 25% of procedure-associated SBP (9). Collectively, these data suggest that Gram-positive SBP is no longer uncommon in 2025.

Across the five cohorts (*n* = 580), Gram-negative bacteria continued to be predominant (RD = 0.15; 95% CI: 0.09–0.20), with *Enterobacteriaceae*, *Klebsiella pneumoniae*, non-fermenters and *Aeromonas* frequently identified. Gram-positive infections such as *Enterococcus*, *Staphylococcus* and *Streptococcus* accounted for 27–54 cases per study. Our findings aligned with contemporary regional data from North America and the Middle East (4, 22), though variations in prophylaxis use and procedural rates remain important contextual modifiers.

Gram-negative infections were associated with an increased death rate (pooled RR = 1.10, 95% CI: 1.01–1.20), consistent with previous cohorts and meta-analyses of observational studies, although the magnitude of our effect was smaller likely attributable to our adjustment for the severity of liver disease. MDR risk was close to threefold higher in Gram-negative bacteria (RR = 2.94, 95% CI: 1.87–4.64), consistent with prior reports (15, 23), and supported by data from Southeast Asia demonstrating high prevalence of ESBL and related mortality (24, 25). Recent antimicrobial resistance surveillance reports from regional and global systems, including WHO GLASS, EARS-Net, CHINET, and the U.S. CDC AR Lab Network, which consistently demonstrate increasing rates of ESBL-producing and carbapenem-resistant Enterobacteriaceae, supporting our finding of disproportionately higher MDR prevalence among Gram-negative pathogens in cirrhotic abdominal infections. However, MDR definitions varied across studies, and our restricted analysis using uniform definitions showed a similar but slightly attenuated effect (RR = 2.76; 95% CI: 1.68–4.52), underscoring the value of standardization.

Clinical translation may be best framed along two axes. The first is the infection phenotype and acuity, Gram-negative predominance was greater in SBP (RD = 0.18; 95% CI: 0.10–0.26) than in non-SBP infections (RD = 0.09; 95% CI: −0.02–0.20), while ICU or ACLF patients exhibited a relative rise in Gram-positive infections and a narrowed mortality gap. Further relation to local ecology is the second axis. Clinical units with elevated *Enterococcus* or MRSA rates may require adapted empiric therapy spectra. These units will systematically use fluoroquinolone prophylaxis, and fluoroquinolone prophylaxis is a risk factor for an increased risk of *Enterococcus* or MRSA with odds ratio 3.94.

These two axes support pragmatic clinical recommendations. Third-generation cephalosporins may still be a reasonable option for empiric therapy of community-acquired SBP in patients without ACLF. For patients with prior antimicrobial prophylaxis, ICU admission, or ACLF, empiric therapy should include coverage for enterococci. Such anti-enterococcal coverage can be achieved with specific antimicrobials, such as piperacillin–tazobactam, or carbapenems with linezolid or vancomycin added in selected cases. Empiric treatment should cover for gram-negative and anaerobic bacteria in healthcare-associated peritonitis or secondary peritonitis. Meanwhile, Gram-positive coverage in these settings should be based on local unit’s antibiogram. Adhering to three critical management strategies may further improve patient survival beyond antimicrobial choice: early blood and ascitic fluid cultures after infection is suspected, de-escalated antimicrobial therapy by 48–72 h in the wake of definitive culture and susceptibility data, and albumin-based fluid therapy for patients with SBP.

Study-level context (Table 7) helps clarify applicability: four studies enrolled SBP exclusively; ICU proportions ranged 10–22%; prophylaxis exposures were 15–25%; and MDR definitions varied between uniform three-class criteria and pathogen-specific additions (ESBL, CRE, MRSA).

**Table 7:** Study-level baseline characteristics and methodological details of included cohorts.

Study (author, year)	Infection type proportions	ICU enrollment	Fluoroquinolone prophylaxis exposure rate	MDR definition
Kim et al. (6), 2016	SBP: 77 (100%); non-SBP: 0 (0%)	16 (20.8%)	14.3% (rifaximin use within 30 days before SBP diagnosis)	Gram-negative: resistance to ≥3 antimicrobial classes + ESBL-producing Enterobacteriaceae
				Gram-positive: resistance to ≥3 antimicrobial classes + MRSA/VRE
Al-Ghamdi et al. (20), 2019	SBP: 200 (100%); non-SBP: 0 (0%)	44 (22.0%)	83.0% (fluoroquinolones as secondary SBP prophylaxis)	Gram-negative/Gram-positive: resistance to ≥3 antimicrobial classes (no pathogen-specific criteria)
Liu et al. (9), 2020	SBP: 32 (40%); Non-SBP: 48 (60%)	6 (7.5%)	Not specified (invasive procedures reported as OR = 4.99 for infection risk)	Gram-negative: ESBL-producing Enterobacteriaceae + carbapenem-resistant Enterobacteriaceae (CRE)
	(Non-SBP: biliary sepsis, post-procedural peritonitis)			Gram-positive: MRSA + VRE + resistance to ≥3 antimicrobial classes
Furey et al. (21), 2023	SBP: 88 (100%); non-SBP: 0 (0%)	19 (21.6%)	31.0% (ciprofloxacin/trimethoprim-sulfamethoxazole for SBP prophylaxis)	Gram-negative/Gram-positive: resistance to ≥3 antimicrobial classes (including ESBL for Gram-negative, MRSA/VRE for Gram-positive)
Oliveira et al. (7), 2016	SBP: 139 (100%); non-SBP: 0 (0%)	24 (17.3%)	16.0% (quinolone-based prophylaxis before index admission)	Gram-negative/Gram-positive: multiresistant strains (ESBL-producing Enterobacteriaceae, MRSA, VRE, *Pseudomonas aeruginosa*, etc.)

Our ICU-focused findings further suggest that host failure, rather than pathogen genus, is the primary driver of mortality. ICU patients had mean MELD 21.9, consistent with ACLF grade 2–3, and 30-day mortality of 45%. The mortality difference between Gram-negative and Gram-positive infections was small (RD = 0.06; *p* = 0.41). Vasopressor use—29% in ICU versus 2% in wards—was a stronger predictor of death than genus (HR 2.86; *p* = 0.01) (21). These patterns align with Ndomba et al.’s (26) ICU synthesis, where ACLF (CLIF-SOFA ≥6) and septic shock (HR 3.2) outweighed pathogen type (HR 1.1).

For SBP specifically, ACLF appears to be a dominant prognostic factor. A dedicated cohort of 69 SBP patients reported ACLF in 58% of cases, with markedly higher mortality in ACLF-1 (57.7%) and ACLF-3 (100%) compared with non-ACLF (27.6%). Higher CLIF-SOFA scores independently predicted 28- and 90-day mortality. These data complement our subgroup showing 42–45% Gram-positive SBP prevalence in ICU/prophylaxis-exposed settings and support the use of anti-enterococcal coverage in ICU or ACLF-associated SBP.

Antimicrobial stewardship remains a key consideration. Empiric therapy should be stratified by SBP vs. non-SBP and ICU/ACLF vs. ward, refined by local *Enterococcus* and MRSA epidemiology, and narrowed promptly with culture data. Fluoroquinolone prophylaxis—associated with increased Gram-positive SBP (34.7–42%; odds ratio 3.94)—should be reserved for the highest-risk patients. Host-directed therapy (albumin for SBP, early vasopressors for ICU patients) is clinically important, given that ACLF and shock dominate mortality risk.

Limitations include the small number of included studies, incomplete reporting of confounders, variability in MDR definitions, language restrictions, missing prophylaxis data, and limited power for publication bias assessment. GRADE assessment classified MDR evidence as Low–Moderate and mortality/prevalence evidence as Moderate due to imprecision from small sample sizes.

Future research should incorporate standardized MDR definitions, comprehensive data on confounders, prophylaxis exposure, and larger subgroups of ICU and ACLF. Randomized trials of phenotype-tailored empiric therapy and investigations into the mechanisms underlying Gram-negative mortality and resistance would help further refine management strategies.

## 5 Conclusion

This review indicates that Gram-negative bacteria still predominate in cirrhotic abdominal infections, and are associated with greater mortality and MDR rates; however, the clinical applicability is significantly different among subgroups. The clearest evidence of Gram-negative predominance is in SBP (RD = 0.18), justifying Gram-negative–directed empiric therapy for community-acquired SBP in ward patients with low prophylaxis exposure. Conversely, those with high fluoroquinolone prophylaxis and frequent invasive procedures, report anywhere from 42 to 45% of SBP caused by Gram-positive organisms, suggesting that early anti-enterococcal coverage is needed on a routine basis until the clinical spectrum is defined by cultures. Non-SBP infections are more Gram-positive, which points to the need for initial regimens that are more balanced. None of these trend echoes the need for differentiation by infection type, care setting, and local ecology rather than “cirrhotic abdominal infection” as a monolithic entity.

For ICU and ACLF patients, our results and others suggest that host failure—ACLF Grade 2–3, septic shock, vasopressor support, and RRT—may have a greater impact on mortality than pathogen genus. Consequently, broad initial coverage might be justified, but early de-escalation remains as critical as ever to minimize antimicrobial pressure, because MDR is unlikely to significantly influence prognosis independently in this context of high severity. Taken together, these subgroup-specific observations also advance the prospect of empiric therapy that is safer, and ecologically aware, and phenotype-tailored stewardship.

## Data Availability

The original contributions presented in the study are included in the article/supplementary material, further inquiries can be directed to the corresponding author.
